# Case Report: Lipoma of the Tuber Cinereum Mimicking a Pituitary Gland Abnormality in a Girl With Central Precocious Puberty

**DOI:** 10.3389/fendo.2021.766253

**Published:** 2021-10-11

**Authors:** Claudio Giacomozzi, Lisa Nicolì, Carlo Sozzi, Enrico Piovan, Mohamad Maghnie

**Affiliations:** ^1^ Unitá Operativa Complessa (U.O.C.) of Paediatrics, Department of Maternal and Child Health, Carlo Poma Hospital, ASST-Mantova, Mantova, Italy; ^2^ U.O.C. of Neuroradiology, Department of Health Services, Carlo Poma Hospital, ASST-Mantova, Mantova, Italy; ^3^ Department of Paediatrics, IRCCS Istituto Giannina Gaslini, Genova, Italy; ^4^ Department of Neuroscience, Rehabilitation, Ophthalmology, Genetics, Maternal and Child Health, University of Genova, Genova, Italy

**Keywords:** lipoma, precocious puberty, MRI, pituitary gland—abnormalities, case report

## Abstract

**Introduction:**

Magnetic Resonance Imaging (MRI) is the best approach to investigate the hypothalamic-pituitary region in children with central precocious puberty (CPP). Routine scanning is controversial in girls aged 6-8 year, due to the overwhelming prevalence of idiopathic forms and unrelated incidentalomas. Cerebral lipomas are rare and accidental findings, not usually expected in CPP. We report a girl with CPP and an unusually shaped posterior pituitary gland on SE-T1w sequences.

**Case Description:**

A 7.3-year-old female was referred for breast development started at age 7. Her past medical history and physical examination were unremarkable, apart from the Tanner stage 2 breast. X-ray of the left-hand revealed a bone age 2-years ahead of her chronological age, projecting her adult height prognosis below the mid parental height. LHRH test and pelvic ultrasound were suggestive for CPP. Routine brain MRI sequences, SE T1w and TSE T2w, showed the posterior pituitary bright spot increased in size and stretched upward. The finding was considered as an anatomical variant, in an otherwise normal brain imaging. Patient was started on treatment with GnRH analogue. At a thorough revaluation, imaging overlap with adipose tissue was suspected and a new MRI scan with 3D-fat-suppression T1w-VIBE sequences demonstrated a lipoma of the tuber cinereum, bordering a perfectly normal neurohypophysis. 3D-T2w-SPACE sequences, acquired at first MRI scan, would have provided a more correct interpretation if rightly considered.

**Conclusion:**

This is the first evidence, to our knowledge, of a cerebral lipoma mimicking pituitary gland abnormalities. Our experience highlights the importance of considering suprasellar lipomas in the MRI investigation of children with CPP, despite their rarity, should the T1w sequences show an unexpected pituitary shape. 3D-T2w SPACE sequences could be integrated into standard ones, especially when performing MRI routinely, to avoid potential misinterpretations.

## Introduction

Magnetic resonance imaging (MRI) is the gold standard for investigating the pituitary gland and adjacent structures in children with pituitary related disorders (1), including central precocious puberty (CPP). CPP is defined as breast enlargement in females before the age of 8 years, and as testicular enlargement >4 ml in males before the age of 9 years (2–4). MRI is reliable in discerning idiopathic CPP (more frequent in females) from organic forms (more frequent in males), such as, tuber cinereum hamartomas, germinomas or other rare neoplasia (5–7). Lipomas of the central nervous system are considered to be a form of congenital cerebral malformation rather than proper primary neoplasia or hamartomas (8). They are rare findings both in adults and children, and are mostly discovered incidentally as they are almost always asymptomatic (8). Suprasellar lipomas, which have been described in a small number of CPP patients (9–11), are generally not considered in the differential diagnosis, because of their rarity and because there is no proven correlation between them and CPP pathogenesis. We report a unique case, to our best knowledge, of a lipoma of the tuber cinereum causing an unusual shape to the posterior pituitary gland in a female with CPP, initially considered as an anatomical variant.

## Case Report

**Figure 1:**
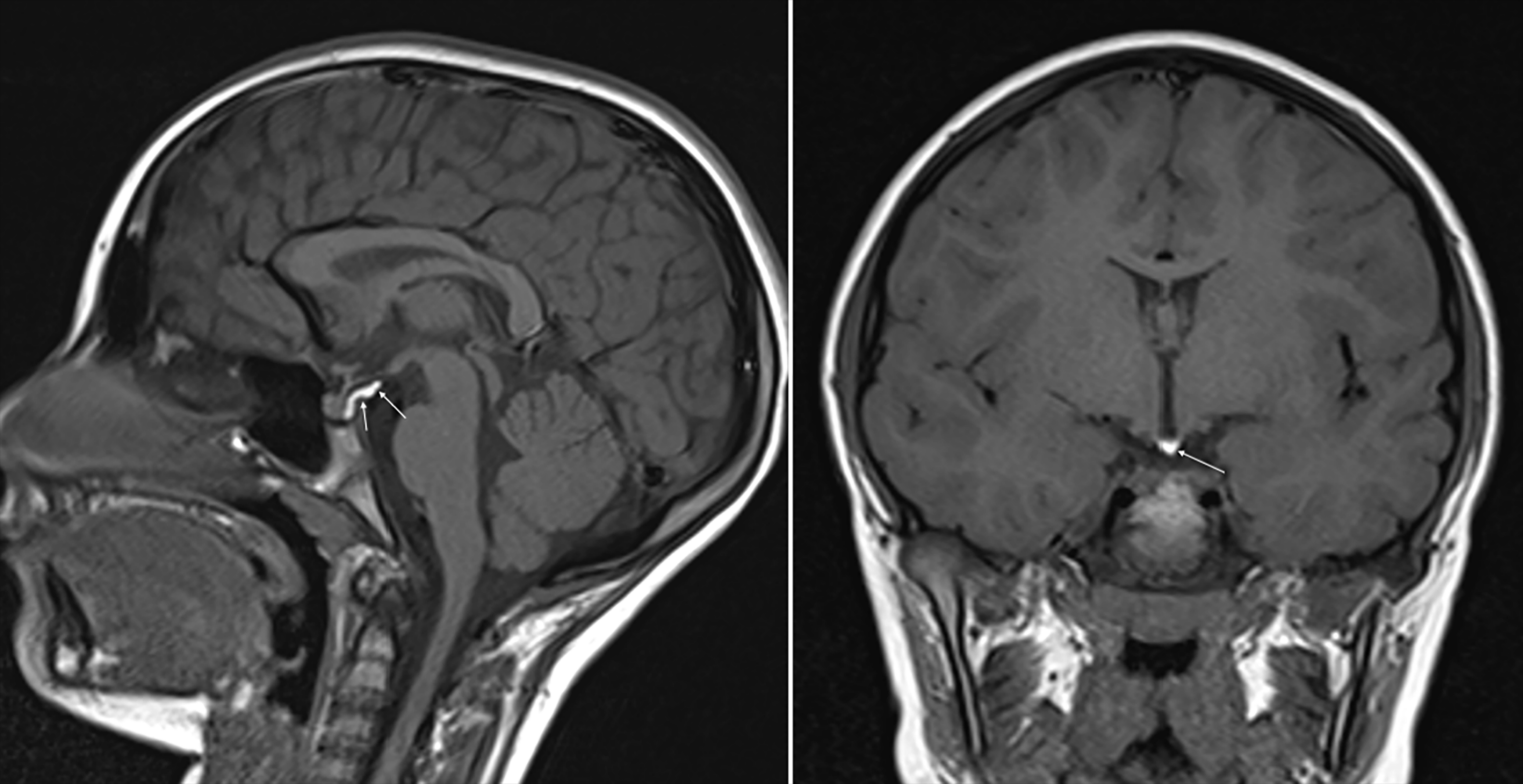
SE T1w sequences (sagittal on the left and coronal on the right) show a posterior bright spot of neurohypophysis increased in size and stretched upward.

**Figure 2:**
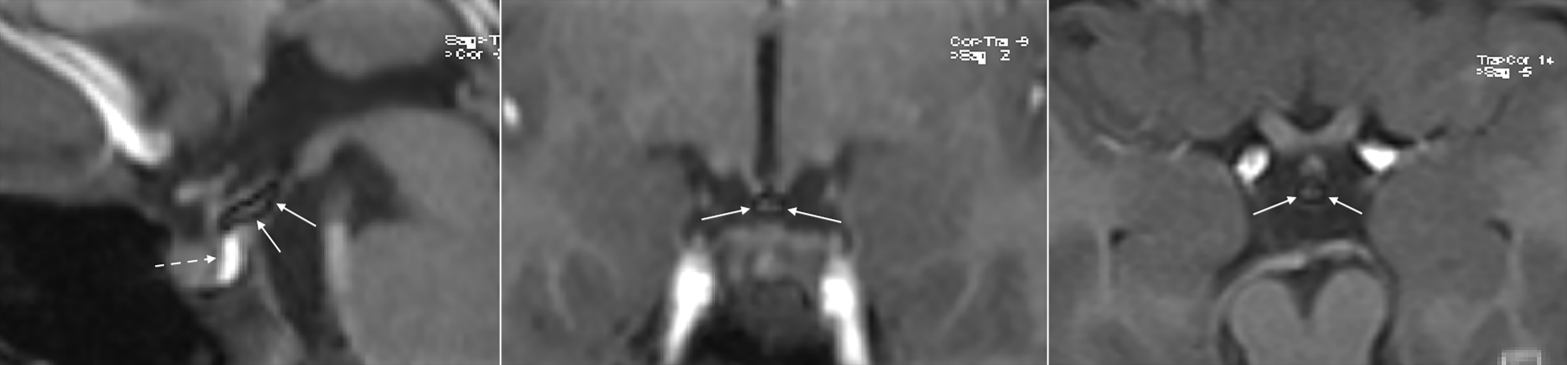
3D Fat Suppression T1w VIBE sequences (sagittal, coronal and axial from left to right) confirm the hypothesis of lipoma of the tuber cinereum, as the upper part of the “unusual pituitary bright spot” disappears, leaving a small hypointense central area most probably representing a fibrosus core (continuous arrows). The neurohypophysis remains unchanged as expected, normal in signal, shape, and size (dotted arrow).

A 7.3-year-old female was referred to the pediatric endocrine outpatient clinic for assessment due to breast development that started at age 7. The patient was born at term by spontaneous delivery without complication, and her past medical history was unremarkable. In the family history paternal grandmother was reported to have menarche at 9 years. Father’s height was normal (170 cm, -1.0 sds), and reliable information about his puberty onset was not available, making firm conclusion on potential CPP in the father impossible. No history of CPP was reported in the maternal branch. After initial examination, which was unremarkable apart from the Tanner stage 2 breast (see **Table 1**), an X-ray of the left-hand was performed and bone age resulted 9.4 years (according to Greulich and Pyle’s standards), 2 years ahead of her chronological age, projecting her adult height prognosis below the target height range [165 cm (0.41 sds) ± 8.5 cm]. Therefore, she underwent luteinizing hormone (LH)-releasing hormone test, which was suggestive for CPP (results are detailed in **Table 1**). Pelvic ultrasound showed increased bilateral ovarian volume and detectable endometrial echo, while uterine fundus-to cervix ratio and uterine length were borderline (see **Table 1**). Routine brain MRI sequences, SE T1w and TSE T2w, showed the posterior pituitary bright spot increased in size and stretched upward (**Figure 1**). The anterior pituitary gland and pituitary stalk were unremarkable, as were the remaining brain structures. At first evaluation, it was suggested that anatomical variation might explain such an uncommon “snake-like” neurohypophysis shape. Further enlargement of breast was reported by the patient. After exhaustive discussion with the patient’s family, therapy with GnRH analogue was started to prevent precocious uterine bleeding and adult short stature (18). However, no similar MRI images were described in the literature, not in girls with CPP, or in patients with other pituitary disorders, or as a normal variation in healthy people. The MRI images were then submitted to four experts in pediatric endocrinology, who confirmed the finding as new. The bright appearance of a structure overlapping the pituitary bright spot, seen with T1w images, was assumed to potentially explain the unusual finding, and the presence of a cerebral lipoma was suggested. A further pituitary MRI scan, with 3D fat suppression T1 VIBE sequences (**Figure 2**), showed a normal posterior pituitary gland both in shape and size, and the presence of a suprasellar lipoma close to the tuber cinereum, and extending down to the neurohypophysis, measuring 1.15 cm x 0.5 cm x 0.35 cm in craniofacial, transverse and antero-posterior dimensions, respectively. The patient and family were informed about the benign nature of lipomas, the lack of evidence about a causal correlation between CPP and suprasellar lipomas, and that MRI follow-up was not recommended unless symptoms occur. Reduction in size of the patient’s breast was noticed after a 6-months period starting GnRH analogue.

**Table 1:** Relevant clinical features and investigations from the episode of care according to timeline.

Age (years)	Clinical features, investigations and results
7	Onset of breast enlargement
7.3	Pubertal staging: B2, PH1, AH1; Height 126.4 cm (0.45 sds); weight 29.4 kg (0.64 sds); BMI 18.4 kg/m2 (0.74 sds), Target Height 165.0 cm (0.41 sds) ± 8.5 cm
7.35	Bone age: advanced at 9.4 yr
7.4	LHRH test: LH basal 0.3 mUI/ml, peak 6.0 mUI/ml (p.v.< 5); FSH basal 1.4 mUI/ml, peak 10.9 mUI/ml
	Estradiol 14 pg/ml (p.v.< 20), TSH 3.01 mcUI/ml (n.r.0.6-4.8), fT4 1.33 ng/dl (n.r.0.97-1.6)
	Pelvic ultrasound: ovarian volume 1.9 ml bilaterally (p.v.< 1.2); uterine fundus-to-cervix ratio = 1:1 (p.v.<1), uterine length 35 mm (p.v. = 34-40), endometrial thickness 1.5 mm (p.v.= not detectable)
7.5	First MRI scan with evidence of unusual neurohypophysis shape
	Start GHRH analogue treatment - Triptorelin 3.75 mg/every 4 weeks
7.6	Repetition of MRI scan confirming the presence of a lipoma of the tuber cinereum

Standard deviation score (SDS) values are expressed according to national references (12). MPH, mid parental height. Bone age has been assessed according to Greulich and Pyle’s references (13), by the automatized BoneXpert software (Visiana, Hørsholm, Denmark). Cut-off of 5 mUI/ml for LH peak after stimulus with Lhrh Ferring 100 mcg/1 mL (Ayerst Laboratories Inc., Philadelphia, USA) was considered according to the current evidence in literature (14, 15). In brackets are the normal ranges (n.r) for thyroid function tests. Pelvic ultrasound was performed by an experienced gynecologist, in brackets are reported prepubertal values (p.v) according to current literature for ovarian volume (16) and for remaining parameters (17).

## Discussion

Brain MRI is used in CPP work-up to exclude rare neoplasia (such as hamartomas, astrocytomas, or other, even rarer neoplasia) underlying the precocious activation of the hypothalamic-pituitary-gonadal axis (4). MRI is considered mandatory for females younger than 6 years (18–20) and all males with CPP to identify possible organic causes (18, 20, 21). Conversely, in females with CPP aged 6–8 years, MRI is controversial, as idiopathic forms are almost all the cases in this age group (6, 18). Nevertheless, some authors still support MRI screening of all females with CPP, based on the possibility of finding rare malignancies at any age. The rationale for this approach is to not risk a possible late cancer diagnosis (7), particularly as clinical and biochemical parameters cannot be used to distinguish females with normal MRI scans from those with pathological scans (5–7). Furthermore, the proportion of incidentalomas unrelated to CPP is estimated to be around 10% (6, 7), which may generate interpretation uncertainty for radiologists and clinicians. In MRI scanning a girl aged 6–8 years with CPP, the frequency of incidentaloma needing a correct interpretation will likely be greater than the frequency of readily identifiable neoplastic forms.

Brain lipomas are, classically, accidental findings during brain imaging (CT or MRI scans) originally arranged for other reasons. Since 1987 many theories have been postulated to explain the pathogenesis of intracranial lipomas. Nowadays the most accepted considers them as congenital malformations resulting from abnormal persistence and abnormal differentiation of the meninx primitiva during the development of the subarachnoid cisterns (8). Lipomas are almost always completely asymptomatic, except in those rare cases when their excessive growth or specific position leads to neurologic signs such as seizures, headaches and/or behavioral disturbances (22, 23). Lipomas can be ubiquitous within the central nervous system, but the majority are described as supratentorial and along midline structures, especially in children. Infratentorial lipomas are more likely to be symptomatic (22). Lipomas are most often found near the corpus callosum (67% in children), less frequently in the cisterna ambiens, quadrigemina and suprasellar (8% in children) (8, 22). For suprasellar lipomas, the tissue is usually found adjacent to the floor of the third ventricle immediately behind the infundibulum of the pituitary gland, in close proximity to the hypothalamus, tuber cinereum and mammillary bodies.

Intracranial lipomas can be tubulonodular or curvilinear in shape (24), and they are often associated with midline anomalies, the most frequent of which is agenesia/dysplasia of the corpus callosum (up to 50% in some case studies) (25). Our patient did show a curvilinear lipoma in the suprasellar cistern, close to the tuber cinereum and contiguous to the posterior pituitary gland, without any midline deformities. Lipomas show an adipose tissue-like signal with a higher signal rate in SE-T1w images and a moderately high signal rate in TSE-T2w images. The posterior pituitary gland is characterized by a bright signal on SE-T1w sequences on MRI. This superimposition of the hyperintense signals in the sagittal and coronal SE-T1w sequences of the neurohypophysis and lipoma (**Figure 1**) led us, initially, to the misleading interpretation that our patient carried an anatomic variant of the neurohypophysis never observed previously. 3D-FAT-SAT T1w VIBE sequences, performed at a later date confirmed, the presence of a lipoma of the tuber cinereum and a normal pituitary gland (**Figure 2**). An obvious point that arises is whether the presence of the lipoma could have been suspected in the first scan, avoiding additional MRI examinations and cumbersome to the patient and family.

**Figure 3:**
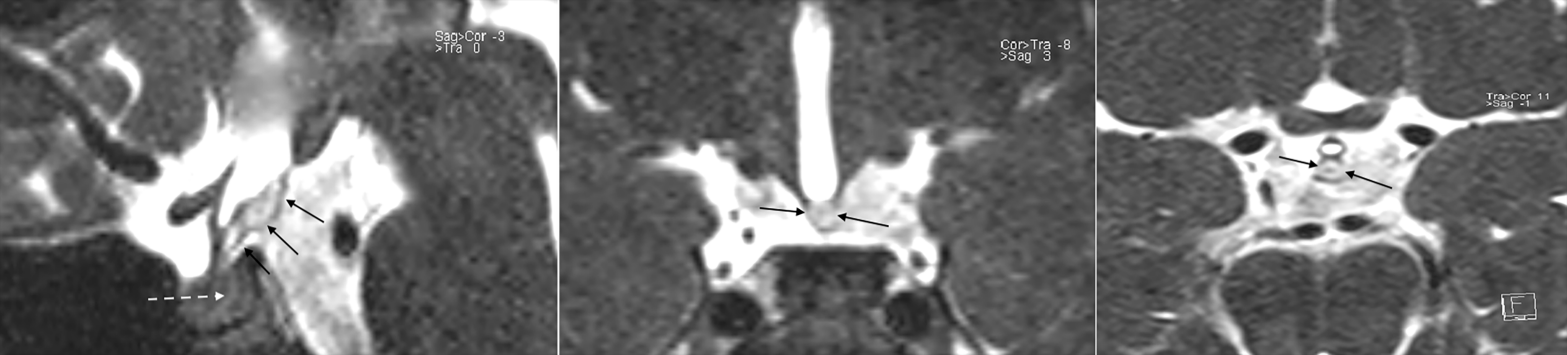
3D T2w SPACE sequences (sagittal, coronal and axial from left to right) show a high signal posterior to the tuber cinereum and poor contrast of the other tissues (continuous arrows); meaning the presence of a tissue in the suprasellar cistern, posterior to the tuber cinereum, different from neurohypophysis which remain hypointense as expected (dotted arrow). Fat has high signal on this sequence, suggesting being a lipoma of the tuber cinereum. The careful consideration of these sequences at the first MRI scan, furtherly to routine sequences, could have driven promptly to the 3D Fat Suppression T1w VIBE sequences for confirmation.

On review of the first scan images, 3D-T2w SPACE sequences had been acquired but not enough considered to evaluate the unusual finding in the SE-T1w sequences. 3D-T2w SPACE sequences showed the lipoma conserved a moderately hyperintense signal, differing from the neurohypophysis, which appeared normally hypointense (**Figure 3**). The acronym “SPACE” stands for “sampling perfection with application optimized contrast using different flip angle evolutions” (SPACE, Siemens Healthcare, Erlangen, Germany). Using 3D-SPACE, which is a TSE variant, it is possible to obtain heavily T2w images as well as T1w, T2w, or FLAIR images without gradient-based artifacts. In addition to providing excellent spatial resolution without interslice gaps, and with reduced flow sensitivity, which could be reconstructed in any desired plane, the use of a variable flip angle further decreases the acquisition time and reduces specific absorption rate (SAR). Because of high T2/T1 ratio, water and fat have high signal on this sequence. 3D-T2w SPACE provides excellent contrast between cerebrospinal fluid and other structures. Finally, gadolinium-based contrast agents, no longer routinely used in the MRI scanning of patients with CPP subsequently the evidence of intracranial gadolinium deposits following intravenous administration (26), would not have provided further useful information. According to our experience and instruments (Magnetom Aera 1.5 T, Siemens Healthcare, Erlangen, Germany), 3D-T2w SPACE images acquisition takes less time than contrast-enhanced T1w sequences on sagittal and coronal planes (4 minutes and 44 seconds vs 7 minutes). Valuable is also the high-quality standard offered by 3D-T2 SPACE images on the assessment of pituitary stalk, which would be more challenging otherwise (27). Replacement of contrast-enhanced T1w sequences with 3D-T2w SPACE, on a routine basis, allows savings in terms of time and money (gadolinium contrast costs), keeping high the sensitivity and specificity on the whole pituitary anatomy.

Our report is not the first describing a cerebral lipoma in CPP. Osteolipomas of the tuber cinereum have been described in females in both 2002 and 2012 (9, 10), and a suprasellar lipoma in a male in 2005 (11), all of whom presented only with CPP. The age at presentation was similar to our patient for two of them, 7.6 (10) and 7 (11) years. While, the age of puberty onset was not detailed in the first case (9), although earlier than 8 years. The suprasellar lipoma in the male was considered a chance association to CPP (11), while the osteolipomas of the tuber cinereum were suspected to be causative, since in one case, surgical excision of the suprasellar lipoma led to breast reduction and normalization of endocrine biomarkers in the patient (9), while in the other report, the authors postulated that CPP could have been triggered by compression of the pituitary stalk by the osteolipoma (10). Anyway, evidence for a causative correlation is not currently available, and conservative management has been demonstrated the most appropriate approach in asymptomatic intracranial lipomas. Furthermore, MRI follow-is not recommended, as majority of incidental lipomas are stable over time (28, 29). In contrast to our patient, in all previous published cases, the lipomas were described as separate from the neurohypophysis, and did not create interpretational difficulties. The unique appearance, and the extremely rare association of suprasellar lipomas in children with CPP, might have contributed to the delay in the correct differential diagnosis in our patient. Other pituitary region masses with a high intrinsic SE-T1w signal are limited to dermoid and epidermoid cysts (30), or mature and immature teratomas (31).

Our experience highlights the importance of considering suprasellar lipomas in the MRI investigation of children with CPP, despite their rarity. Furthermore, we suggest the (routinely) use of 3D-T2w SPACE sequences to avoid potential misinterpretations should the T1w sequences show an unexpected pituitary bright spot shape, in way to promptly complete the examination with 3D-FAT suppression T1w VIBE sequences.

## Patient Perspective

The repetition of an MRI, in general, may generate in patients, and their family, further anxiety about own health, and uncertainty in the information received by the medical team. Therefore, every single brain MRI, especially in children, should carefully performed to be conclusive on the findings reported, to the best of moderns imaging techniques. The implementation of 3D-T2w SPACE sequences in a standard MRI scan, as demonstrated in this case, allow to point to the correct interpretation of abnormal neurohypophysis shapes from the first scan. Its routinely use in patients with central precocious puberty positively impacts on the medical management, avoiding potential confounding messages to the families, due to a significant percentage of cerebral incidentalomas, even in those as rare as suprasellar lipomas.

## Data Availability Statement

The original contributions presented in the study are included in the article/**Supplementary Material**. Further inquiries can be directed to the corresponding author.

## Ethics Statement

Written informed consent was obtained from the minor(s)’ legal guardian/next of kin for the publication of any potentially identifiable images or data included in this article.

## Author Contributions

CG managed the endocrine investigation of the patient, drafted the manuscript, and supervised the entire work. LN, CS, and EP performed the MRI scans and revised the manuscript for the MRI imaging details. MM postulated the presence of a cerebral lipoma and revised the entire manuscript. All authors contributed to the article and approved the submitted version.

## Conflict of Interest

The authors declare that the research was conducted in the absence of any commercial or financial relationships that could be construed as a potential conflict of interest.

## Publisher’s Note

All claims expressed in this article are solely those of the authors and do not necessarily represent those of their affiliated organizations, or those of the publisher, the editors and the reviewers. Any product that may be evaluated in this article, or claim that may be made by its manufacturer, is not guaranteed or endorsed by the publisher.
