# Supplementary material for: Case Report: Lipoma of the Tuber Cinereum Mimicking a Pituitary Gland Abnormality in a Girl With Central Precocious Puberty
Source: Front Endocrinol (Lausanne). 2021 Oct 11;12:766253. doi: 10.3389/fendo.2021.766253 (PMC8542968; doi:10.3389/fendo.2021.766253)
Supplement: Supplementary file 1 [file DataSheet_1.pdf]

CARE case report guidelines

CARE Checklist of information to include when writing a case report

(cc) BY-NC-ND

✔

Topic	Item	Checklist item description	Reported on Line
**Title**	1	The diagnosis or intervention of primary focus followed by the words "case report"	1-2
**Key Words**	2	2 to 5 key words that identify diagnoses or interventions in this case report, including "case report"	19
**Abstract (no references)**	3a	Introduction: What is unique about this case and what does it add to the scientific literature?	25-30
	3b	Main symptoms and/or important clinical findings	31-41
	3c	The main diagnoses, therapeutic interventions, and outcomes	31-41
	3d	Conclusion—What is the main "take-away" lesson(s) from this case?	42-46
**Introduction**	4	One or two paragraphs summarizing why this case is unique (**may include references**)	50-63
**Patient Information**	5a	De-identified patient specific information.	66-69 and Tab 1
	5b	Primary concerns and symptoms of the patient.	66-69
	5c	Medical, family, and psycho-social history including relevant genetic information	66-69
	5d	Relevant past interventions with outcomes	None
**Clinical Findings**	6	Describe significant physical examination (PE) and important clinical findings.	66-69
**Timeline**	7	Historical and current information from this episode of care organized as a timeline	Tab 1 line 290
**Diagnostic Assessment**	8a	Diagnostic testing (such as PE, laboratory testing, imaging, surveys).	69-91 and Tab 1
	8b	Diagnostic challenges (such as access to testing, financial, or cultural)	None
	8c	Diagnosis (including other diagnoses considered)	88-90
	8d	Prognosis (such as staging in oncology) where applicable	81
**Therapeutic Intervention**	9a	Types of therapeutic intervention (such as pharmacologic, surgical, preventive, self-care)	74 and Tab 1
	9b	Administration of therapeutic intervention (such as dosage, strength, duration)	Tab 1 line 287
	9c	Changes in therapeutic intervention (with rationale)	None
**Follow-up and Outcomes**	10a	Clinician and patient-assessed outcomes (if available)	90-91
	10b	Important follow-up diagnostic and other test results	86-90
	10c	Intervention adherence and tolerability (How was this assessed?)	83-84
	10d	Adverse and unanticipated events	None
**Discussion**	11a	A scientific discussion of the strengths AND limitations associated with this case report	94-161
	11b	Discussion of the relevant medical literature **with references**.	94-161
	11c	The scientific rationale for any conclusions (including assessment of possible causes)	144-151 / 134-142
	11d	The primary "take-away" lessons of this case report (without references) in a one paragraph conclusion	157-161
**Patient Perspective**	12	The patient should share their perspective in one to two paragraphs on the treatment(s) they received	164-172
**Informed Consent**	13	Did the patient give informed consent? Please provide if requested	Yes ☒ No ☐
